# Association between *MGMT* Promoter Methylation and Risk of Breast and Gynecologic Cancers: A Systematic Review and Meta-Analysis

**DOI:** 10.1038/s41598-017-13208-3

**Published:** 2017-10-06

**Authors:** Ru Chen, Yonglan Zheng, Lin Zhuo, Shengfeng Wang

**Affiliations:** 1 National Cancer Center/Cancer Hospital, Chinese Academy of Medical Sciences and Peking Union Medical College, Beijing, China; 2 Department of Medicine, University of Chicago, Chicago, IL USA; 3 Department of Epidemiology & Bio-statistics, School of Public Health, Peking University Health Science Center, Beijing, China

## Abstract

The role of the promoter methylation of *O* ^6^-methylguanine-DNA methyltransferase (*MGMT*) remains controversial for breast and gynecologic cancers. We conducted a meta-analysis to assess the association between hypermethylation of *MGMT* promoter and the risk of breast and gynecologic cancers. A comprehensive search was conducted in PubMed and Embase electronic databases up to 19th August 2017 for studies about the association between *MGMT* promoter hypermethylation and breast and gynecologic cancers. A total of 28 articles including 2,171 tumor tissues and 1,191 controls were involved in the meta-analysis. The pooled results showed that *MGMT* promoter methylation status was significantly associated with an increased risk of breast and gynecologic cancers (OR = 4.37, 95% CI: 2.68–7.13, *P* < 0.05). The associations were robust in subgroup analysis based on ethnicity, cancer type, methylation detection method, and control source. This meta-analysis indicated that *MGMT* hypermethylation was significantly associated with the risk of breast and gynecological cancers, and it may be utilized as a valuable biomarker in early diagnostics and prognostication of these cancers. Further efforts are needed to identify and validate this finding in prospective studies, especially in situation with new methylation testing methods and samples from plasma circulating DNA.

## Introduction

Malignant diseases of the breast and genitals are the most common cancers in women worldwide, and about 2.8 million new cases and 1.0 million cause-specific death each year^1^. Breast cancer ranks first with 25.5% (1.7 million cases) of all incident cancers, and the genitals (corpus uteri, cervix uteri and ovary) accounts for 16.5% (1.1 million cases) of them. It has generally been accepted that the late diagnosis of breast and gynecologic cancers is a serious global problem, which makes treatment less likely to succeed and reduces their chances of survival^2,3^. As promoter CpG island hypermethylation is considered to be an early alteration in carcinogenesis and is often present in the precursor lesions of a variety of cancers, DNA hypermethylation might be used as a marker for the early diagnosis of cancer^4^.

*O* ^6^-methylguanine-DNA methyltransferase (*MGMT*), is a widely expressed DNA repair gene that plays a crucial role in repair of DNA damage caused by alkylating agents^5,6^. Epigenetic silencing via hypermethylation of specific promoter CpG island is regarded as one of the causes for loss of *MGMT* activity in tumor tissues^7^. It has been suggested that loss of *MGMT* is associated with increased carcinogenic risk and increased sensitivity to therapeutic methylating agents^5^. Although the exact role of *MGMT* promoter methylation in malignant transformation and carcinogenesis remains unrevealed completely, it might be a good biomarker candidate for early cancer detection^5^. The hypermethylation of CpG islands is relatively rare in normal cells, thus the detection of methylated DNA in bodily fluids can be promising^8^. Several studies have focused on this in other cancers including head and neck squamous cell carcinoma, lung cancer and esophageal cancer^9–11^. Nevertheless, for breast and gynecologic cancers, although many studies have explored the association between their risks and *MGMT* promoter hypermethylation, the results remain inconsistent^12–39^. A possible reason to explain the noted discrepancies in results is the inadequate statistical power of the individual studies, especially for relatively rare types (e.g. vaginal cancer and vulvar cancer). Due to breast cancer and gynecologic cancer generally share several common risk factors, such as reproductive history and *BRCA1/2* mutations, it is usually adapted to explore or summarize their associated factors together in a few studies or in clinical resource (such as Physician Data Query)^40–43^. Therefore, we conducted this systematic review and meta-analysis to assess the association between hypermethylation of *MGMT* promoter and the risk of breast and gynecologic cancers.

## Results

### Study selection

**Figure 1:**
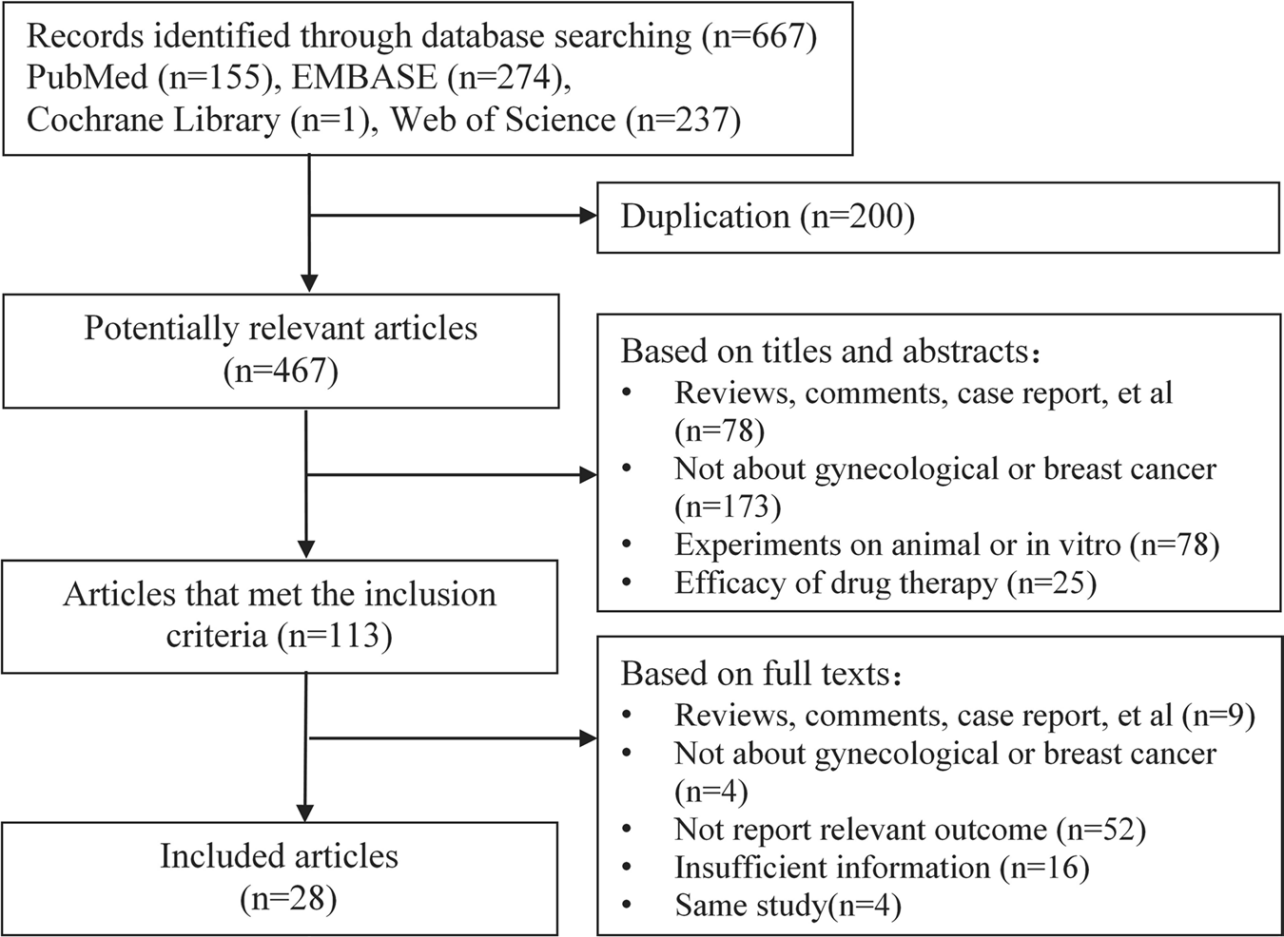
Flow diagram of the results of the search strategy.

The selection flow of studies was summarized in Fig. 1. The initial search identified 429 studies on breast and gynecological cancers risk and/or clinical outcome assessment for *MGMT* hypermethylation. According to the inclusion criteria, 28 articles were included in our meta-analysis. One article reported two cancers^16^ separately and thus was divided to two studies.

### The characteristics of included studies

All the eligible studies were issued in English. In total of 2,171 cases and 1,191 controls were involved in the pooled analyses. The publication year of selected studies ranged from 2001 to 2015. All studies focused on Caucasians or Asians except for two studies in the USA^14,31^ that also included black and other mixed populations. Table 1 presents the primary characteristics and quality assessment of the included studies. The quality of primary studies assessed by NOS showed that most studies (25 out of 29) were rated as “high quality”.

**Table 1:** Baseline Characteristics of Eligible studies

Author	Year	Country	Ethnicity	Diagnosis	Methylation detection method^c^	Sample type		Control source^d^	NOS Score
						case	control		
Virmani^36^	2001	USA	Caucasian	cervical cancer	MSP	tissue	blood and buccal epithelial cells	H	6
Zemlyakova^39^	2003	Russia	Caucasian	breast cancer	MSP	tissue	tissue and blood	H	6
Yang^29^	2004	China	Asian	cervical cancer	MSP	tissue	tissue and blood	A	7
Kang^19^	2005	Korea	Asian	cervical cancer	MSP	tissue	tissue	H	6
Lin^23^	2005	Korea	Asian	cervical cancer	MSP	tissue	tissue	H	5
Makaria^38^	2005	USA	Caucasian	ovarian cancer	MSP	tissue	tissue	H	5
Kekeeva^20^	2006	Russia	Caucasian	cervical cancer	MSP	tissue	exfoliated cells and tissue	A and H	8
Furlan^16^	2006	Italy	Caucasian	endometrial cancer	MSP	tissue	tissue	A	8
		Italy	Caucasian	ovarian cancer	MSP	tissue	tissue	A	8
Suehiro^27^	2008	Japan	Asian	endometrial cancer	MSP	tissue	tissue	H	7
Iliopoulos^18^	2009	USA, Greece	Caucasian	cervical cancer	MethyLight	tissue	tissue	H	6
Flatley^15^	2009	UK	Caucasian	cervical cancer	MSP	exfoliated cells	exfoliated cells	H	6
An^31^	2010	USA	Mixed^a^	ovarian cancer	MSP	tissue	tissue	H	6
Kim^21^	2010	Korea	Asian	cervical cancer	MSP	exfoliated cells	exfoliated cells	H	6
Muggerud^24^	2010	Norway	Caucasian	breast cancer	Pyrosequencing	tissue	tissue	H	6
Sharma^26^	2010	India	Asian	breast cancer	MSP	tissue	tissue	A	8
Guerrero^17^	2011	Spain	Caucasian	vulvar cancer	MSP	tissue	tissue	A	8
Dong^35^	2011	Korea	Asian	cervical cancer	MSP	tissue	tissue	H	7
Roh^25^	2011	Korea	Asian	ovarian cancer	MSP	tissue	tissue	H	6
Chmelarova^33^	2012	Czech	Caucasian	ovarian cancer	MS-MLPA	tissue	tissue	H	7
Sun^28^	2012	China	Asian	cervical cancer	MSP	exfoliated cells	exfoliated cells	H	8
Alkam^12^	2013	Japan	Asian	breast cancer	MSP	tissue	tissue	H	6
Brait^14^	2013	USA, Mexico	Mixed^b^	ovarian cancer	QMSP	tissue	tissue	H	7
Klajic^22^	2013	Norway	Caucasian	breast cancer	Pyrosequencing	tissue	tissue	H	6
de Groot^34^	2014	Netherland	Caucasian	breast cancer	MSP	tissue	tissue	H	6
Banzai^13^	2014	Japan	Asian	cervical cancer	MSP	tissue	tissue	H	6
Shilpa^37^	2014	India	Asian	ovarian cancer	MSP	tissue	tissue	H	6
Spitzwieser^30^	2015	Austria	Caucasian	breast cancer	MS-HRM	tissue	tissue	H	5
Asiaf^32^	2015	India	Asian	breast cancer	MSP	tissue	tissue	A	8

aNon-Hispanic white, African American, Mexican American and others

bCaucasian, African-American, Hispanic and others

cMSP, methylation-specific polymerase chain reaction; QMSP, real-time quantitative MSP; MS-HRM, methylation-sensitive high-resolution melting analysis; MS-MLPA, methylation-specific multiplex ligation-dependent probe amplification.

dA: Autologous, H: Heterogeneous

### Meta-analysis

**Figure 2:**
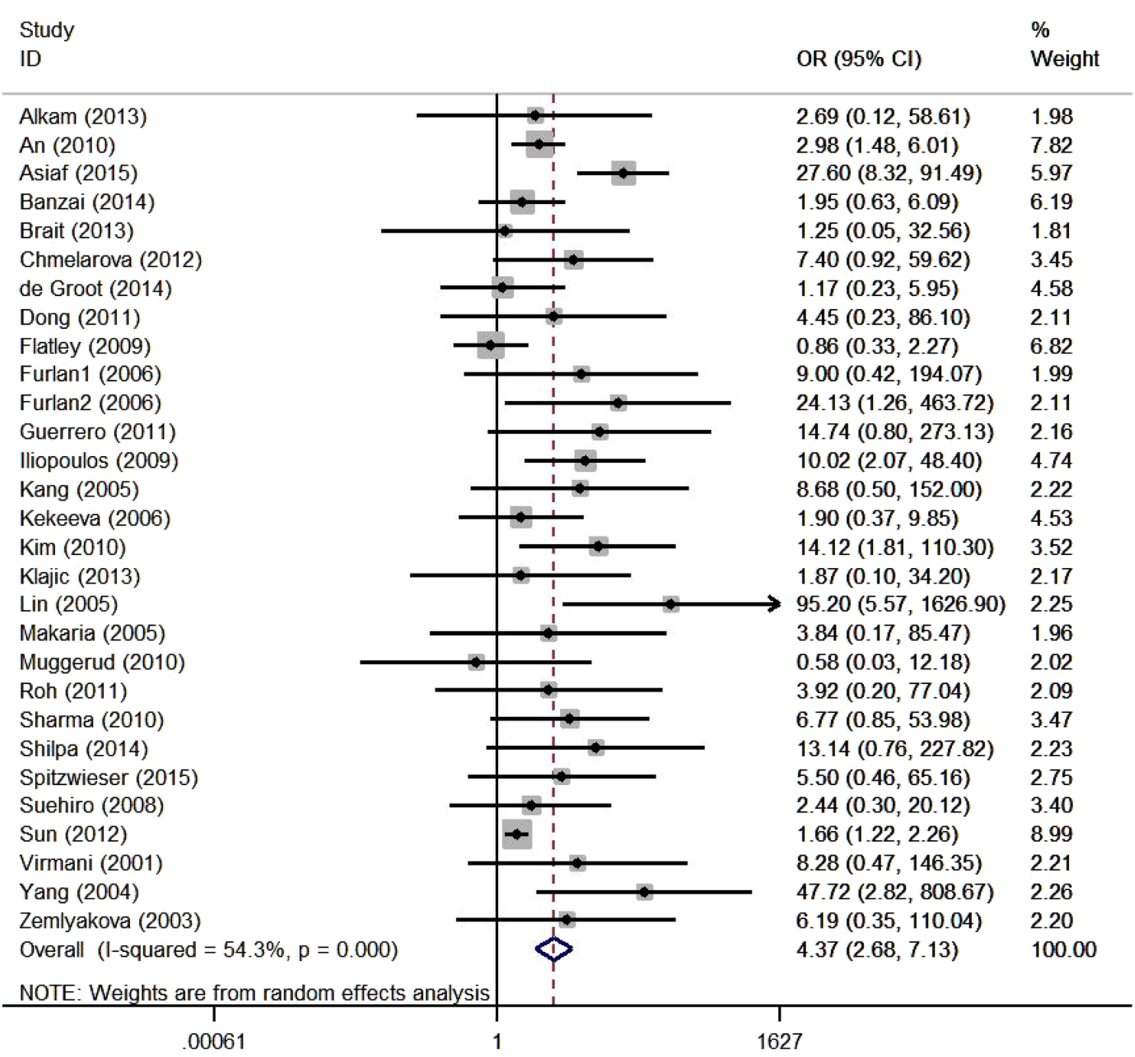
Forest plot of *MGMT* promoter methylation and risk of breast and gynecological cancers in women.

The combining result of the association of *MGMT* promoter hypermethylation with risk of breast and gynecological cancers was shown in Fig. 2. The random effect model was employed due to the significant heterogeneity among the included studies (I^2^ = 54.3%, *P* < 0.05). The pooled results showed that *MGMT* promoter methylation status was significantly associated with an increased risk of breast and gynecological cancers in women (OR = 4.37, 95% CI: 2.68–7.13, *P* < 0.05).

### Subgroup analysis

We performed subgroup analysis to evaluate the source of the heterogeneity according to ethnicity, cancer type, methylation detection method, and control source (Table 2). No significant differences were observed in subgroup analysis based on neither ethnicity nor cancer type. Most studies used MSP to detect the frequency of *MGMT* promoter methylation, other methods including pyrosequencing, QMSP, MS-MLPA, MS-HRM and MethyLight were classified as non-MSP group. The ORs were 4.56 (95% CI: 2.62–7.95, *P* < 0.05) in the MSP group under random effects model, and 4.60 (95% CI: 1.78–11.85, *P* < 0.05) in the non-MSP group under the fixed-effects model. With regard to the control source, one study^20^ had both autologous and heterogeneous samples as control and was divided into two studies. Three studies were excluded since they included blood sample as controls. The pooled ORs in heterogeneous and autologous tissue group were overlapped under the fixed effects model, and with the value of 3.33 (95% CI: 2.16–5.14, *P* < 0.05) and 11.37 (95% CI: 5.11–25.31, *P* < 0.05), respectively. While in heterogeneous exfoliated cells group, the *MGMT* promoter methylation status was not significantly associated with cancer risk with a pooled OR of 1.83 (95% CI: 0.83–4.06, *P = *0.136).

**Table 2:** Subgroup analysis of the association between *MGMT* promoter methylation and risk of breast and gynecological cancers in women

Subgroup	No. of studies	Heterogeneity		Model selected	OR (95%CI)	*P* value
		I^2^	*P* value			
Total	29	54.3%	<0.05	Random	4.37 (2.68–7.13)	<0.05
Ethnicity						
Asian	13	72.9%	<0.05	Random	6.96 (2.78–17.42)	<0.05
Caucasian	14	20.7%	0.228	Fixed	2.59 (1.52–4.42)	<0.05
Mixed	2	0%	0.608	Fixed	2.87 (1.44–5.69)	<0.05
Cancer						
Breast cancer	8	47.3%	0.066	Fixed	5.96 (2.90–12.27)	<0.05
Ovarian cancer	7	0%	0.741	Fixed	3.70 (2.04–6.71)	<0.05
Cervical cancer	11	65.8%	<0.05	Random	4.14 (1.91–8.99)	<0.05
Endometrial cancer	2	0%	0.492	Fixed	3.71 (0.65–21.11)	0.140
Vulvar cancer	1	—	—	—	14.74 (0.80–273.13)	0.071
Methylation detection method^a^						
MSP	23	60.4%	<0.05	Random	4.56 (2.62–7.95)	<0.05
Non-MSP	6	0%	0.561	Fixed	4.60 (1.78–11.85)	<0.05
Control source^b^						
Heterogeneous tissue	17	0%	0.618	Fixed	3.33 (2.16–5.14)	<0.05
Heterogeneous exfoliated cells	4	51.7%	0.102	Random	1.83 (0.83–4.06)	0.136
Autologous tissue	6	30.3%	0.208	Fixed	11.37 (5.11–25.31)	<0.05

aMSP, methylation-specific polymerase chain reaction; Non-MSP, included pyrosequencing, real-time quantitative MSP, methylation-sensitive high-resolution melting analysis, methylation-specific multiplex ligation-dependent probe amplification and MethyLight.

bThree studies were excluded in this subgroup analysis due to their mixed control source. But one study (Kekeeva, 2006) was divided into two because of its two control sources.

### Sensitivity analysis

Sensitivity analysis performed by excluding the “low quality” study^21,23,30,38^ which got an NOS score < 6. The pooled results were not significant changed for random effects model (OR = 3.76, 95% CI: 2.30–6.15, *P* < 0.05), indicating that patients with hypermethylated *MGMT* may have an increased risk in breast and gynecological cancers.

We also took another sensitivity analysis by excluding the study^23^ with the biggest OR outlier in the random effects model with statistical significant finding. The overall OR was changed from 4.37 (95% CI: 2.68–7.13, *P* < 0.05) to 3.97 (95% CI, 2.49–6.35, *P* < 0.05), which demonstrated that the pooled OR was reliable and stable.

### Publication bias

**Figure 3:**
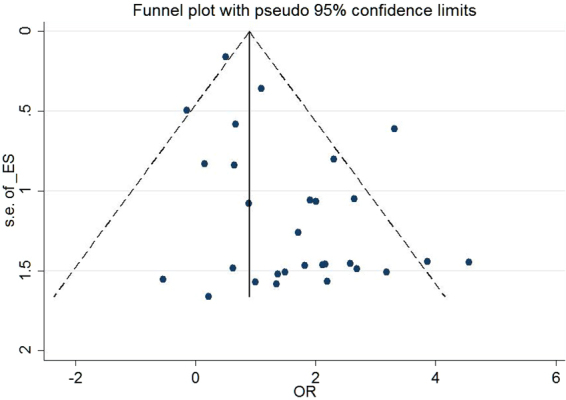
Funnel plot to detect publication bias in the meta-analysis.

Visual inspection of funnel plots and the Egger’s test were used to evaluate the publication bias in our meta-analysis. The funnel plot displayed in Fig. 3 appeared asymmetrical and the statistical test showed significant result (Egger’s test *P* < 0.05), suggesting that there might be publication bias due to small-study effects in our study.

## Discussion

To the best of our knowledge, this meta-analysis is the first to comprehensively evaluate the association between *MGMT* promoter methylation status and risk of breast and gynecological cancers in women. A total of 29 studies including 2,171 tumor tissues and 1,191 controls were involved in the meta-analysis. The proportion of *MGMT* promoter hypermethylation ranged from 3.0% to 70.1% (median: 24.8%) in tumor tissues and 0.0% to 36.9% (median: 0.3%) in non-cancerous controls, respectively. Our major finding suggested that *MGMT* promoter hypermethylation had a significantly increased risk in tumor tissues (OR = 4.37, 95% CI: 2.68–7.13) compared with non-cancerous tissues and exfoliated cells.

About 8 of 29 included studies presented significant association between hypermethylation of *MGMT* promoter and risk of breast and gynecological cancers in women^16,18,21,23,28,29,31,32^, whereas all of the remaining suggested no significant relationship^12–17,19,20,22,24–27,30,33–39^. When all studies were pooled into the meta-analysis, cancer risk associated with *MGMT* promoter hypermethylation was significant in breast and gynecological cancers. The result of sensitivity analysis revealed that this association was quite reliable and stable after excluding the study with the largest OR outlier^23^, or excluding four studies with lower quality^21,23,30,38^. Power analysis was also conducted according to our own data. Assuming OR as 4.0 and proportion of *MGMT* promoter hypermethylation among controls as 0.3%, the powers before and after excluding above studies were both vigorous with a value always larger than 80% in corresponding sample size.

Since heterogeneity obviously existed among studies, stratified analyses were also performed based on ethnicity, cancer types, methylation detection methods, and control source. The subgroup analysis suggested that hypermethylation of the *MGMT* gene was associated with the risk of breast and gynecological cancers in almost all these subgroups, except for endometrial cancer and vulvar cancer due to limited samples (<50)^16,18,28^. Although MSP has some defects which prompt researchers to develop novel test methods, such as pyrosequencing, QMSP,

MS-MLPA, MS-HRM and MethyLight^44,45^, it is still generally accepted as the best way to evaluate the methylation status of the *MGMT* promoter^46^. About 4/5 of included studies have used MSP, and no discrepant results between MSP and non-MSP were showed in our study. We acknowledge that we could not refine the non-MSP in further detail due to the limited related studies, which may need further evaluation in future. In addition, the ORs with autologous tissues as control, were not significantly different from that with heterogeneous tissues, but were significantly larger than that compared with heterogeneous exfoliated cells. It might be explained by the known higher methylation proportion of exfoliated cells in normal or intraepithelial lesions (LSIL, HSIL)^21^. In our pooled result, the *MGMT* methylation rate was more than 30% in exfoliated cells but only ranged from 0% to 14% for the adjacent tissues, which also further supported our explanation.

The *MGMT* gene is ubiquitously expressed in different organs and different tumors and MGMT is responsible for removing the alkyl adducts from the DNA molecules^47,48^. If repair of the alkylating lesions does not complete entirely, a G → A transition mutation or a strand break can occur, resulting in oncogene mutations in pre-malignant lesions (e.g. *KRAS* point mutations), or futile cycles of repair that triggers apoptosis (outcome of therapeutic treatment such as Temozolomide in glioblastoma), respectively^47,48^. In addition, it has been reported that *MGMT* gene expression in normal and neoplatic tissues varies and *MGMT* promoter methylation was associated with better suvivial in some cancer types but not all^47^. In the present study, we showed that *MGMT* promoter methylation status was significantly associated with an increased risk of breast and gynecologic cancers, which is consistent with previous studies in head and neck squamous cell carcinoma, lung cancer, glioblastoma, and esophageal cancer^9–11^. These works including ours highlighted the possibility of using *MGMT* promoter methylation status as a biomarker^49^, based on the facts that *MGMT* promoter hypermethylation could occur early in the neoplastic process before the clinical manifestation^29,50,51^, or turn up in normal appearing tissues close to tumors^52,53^. Currently, it has been indicated that hypermethylation of *MGMT* in circulating DNA might serve as a surrogate marker for tumor methylation in invasive ductal breast carcinomas^26^. Therefore, along with the development of different assays for CpGs methylation^44,54^, our finding provided supporting evidence for diagnosis and prognosis of breast and gynecological cancers with obtaining blood samples instead of biopsies.

We believe that this is the first quantitative study to assess the association between hypermethylation of *MGMT* promoter and the risk of breast and gynecologic cancers. Our results are reliable according to the stability and consistency in all subgroup analysis and sensitivity analyses. Neither specific factor nor single study could significantly affect the summarized OR. However, the presented information still should be interpreted with caution because some limitations existed. Firstly, funnel plots and results of Egger’s test in our study showed significant result. The small-study effect presented clearly base on visual assessment, but it’s hard to attribute this effect entirely to publication bias^55^. Nevertheless, publication bias may still exist considering that some studies were excluded due to unavailable information and that studies with negative results often have less chance for publication. Secondly, the lack of the original data limited the further subgroup analysis based on patients’ comorbidity, BMI, lifestyle and other environmental factors, thus, it is still not sure whether *MGMT* promoter hypermethylation is an independent predictive factor. Thirdly, all of the included studies were retrospective, and prospective cohort studies should be required to confirm our conclusion of its predictive value. Fourth, as an association study, it should be noted that although our results indicated the similar positive associations of *MGMT* promoter hypermethylation with different types of cancer, the exact underlying mechanisms might be still diverse in different types of cancer.

To sum up, this meta-analysis indicated that *MGMT* hypermethylation was significantly associated with the risk of breast and gynecological cancers. Consequently, detection of *MGMT* promoter hypermethylation may be utilized as a valuable biomarker in early diagnostics and prognostication of these cancers. However, further efforts are needed to identify and validate this finding in prospective studies, especially in situation with new methylation testing methods and samples from plasma circulating DNA.

## Materials and Methods

### Literature research

A comprehensive search was conducted to identify all eligible publications in PubMed and Embase electronic databases up to 19th August 2017^56^. We used both the medical subject headings (MeSH) and free-text words. Search terms mainly included methylation, *MGMT* and different gynecological cancer including endometrial cancer, ovarian cancer, vulvar cancer, uterine cancer, vaginal cancer, cervical cancer, fallopian tube cancer, as well as breast cancer in women. The references of the retrieved articles and related reviews were also carefully checked to find additional eligible studies. No language or other limits were set during the course of literature search.

### Inclusion and exclusion criteria

A study was included if it met the following criteria: (1) case-control or cohort study design; (2) evaluated the association between the methylation of *MGMT* and risk of gynecological or breast cancer in women; (3) provided sufficient data (the numbers of methylation status in two groups, respectively) for calculating the odds ratio (OR) and it 95% confidence interval (CI). Letters, comments, conference reports, laboratory studies and articles that didn’t present enough data for ORs calculation were excluded.

### Data extraction

Two reviewers independently read the eligible studies. The following items were extracted from each eligible study: surname of first author, publication year, country of the investigation, ethnicity, diagnosis, method for detecting the methylation status, sample type in case and control groups, and methylation distribution. A discussion was carried out to achieve consensus when discrepancy noted.

### Methodological quality assessment

The Newcastle-Ottawa Scale1 (NOS), one of the most commonly used tools for assessing the quality of observational studies in a meta-analysis setting, was employed to evaluate the quality of eligible studies by two investigators independently^57^. It contains three parts: case and control selection, comparability, and exposure. Each of them respectively comprises four, two, and three items. Each item is given 1 point, 9 points in total. The cut point of 6 points was used to distinguish “low quality” (<6 points) and “high quality” (≥6 points). Disagreements between investigators regarding data extraction were resolved through discussion.

### Data analysis

Crude ORs together with their corresponding 95% CIs were calculated to evaluate the association between *MGMT* promoter hypermethylation and risk of breast and gynecological cancers. We used I^2^ statistic and Q test to measure the between-study heterogeneity. If I^2^ < 50% and *P* > 0.1, the heterogeneity was considered mild, and the summary ORs were combined under a fixed-effects model, otherwise a random-effects model were used. The Z test was used to assess the statistical significance of pooled ORs, and two-tailed *P*-values <0.05 were considered significant. Moreover, we performed subgroup analysis based on ethnicity, cancer type, methylation detection method, and control source to explore potential sources of heterogeneity. Sensitivity analysis were also performed by the study with “low quality”, and excluding the study with the OR outlier with statistically significant findings. The Egger’s test and visual inspection of funnel plots were utilized to explore any possible publication bias. All statistical analyses were conducted in STATA 12.0 (Stata Corporation, College Station, Texas, USA).
